# Edema and myocardial necrosis evaluated by cardiac magnetic resonance in acute myocarditis. Relationship with biomarkers and ventricular function

**DOI:** 10.1186/1532-429X-13-S1-P321

**Published:** 2011-02-02

**Authors:** Joao S Marques, Ana G Almeida, Raquel Magalhaes, Doroteia R Silva, Manuel G Varela, Susana Martins, Dulce A Brito, Antonio N Diogo

**Affiliations:** 1 University Hospital Santa Maria, Lisbon, Portugal

## Introduction

Acute myocarditis has a wide spectrum of clinical presentations. Knowledge of temporal and spatial evolution of inflammation and myocardial necrosis and its correlation with the systemic inflammatory markers may help to recognize patients at risk for progression to chronic cardiac dysfunction.

## Purpose

Our objective was to evaluate if serum biomarkers and markers of myocardial necrosis can predict oedema and necrosis assessed by cardiac magnetic resonance (CMR).

## Methods

We included 31 consecutive patients admitted with the diagnosis of acute myocarditis, confirmed by CMR in the presence of the following criteria: a) chest pain and/or heart failure, b) elevated markers of myocardial necrosis, c) normal coronary angiography. Clinical and laboratorial evaluation (high sensitivity CRP, NT-pro-BNP, troponin I, CK, CK-MB, serology and antibodies) was carried out. Patients underwent evaluation by CMR with determination of ejection fraction and wall motion score index (WMS)=(Σ Score of each segment/17). CMR late gadolinium enhancement (LGE) was used to assess necrosis and a necrosis index (IN) was derived (LGE mass/total myocardial mass). Myocardial oedema was assessed visually from T2W sequence images. Oedema (OS) and necrosis (NS) scores were derived in a segmental basis (1=presence, 2=absence in each segment; Σ Score of each segment/17).

## Results

All patients (26 [[Unsupported Character - ♂]], 5 [[Unsupported Character - ♀]], 26.3±12.5 years) showed an increase of at least one of the markers of necrosis (100% with elevated troponin levels (16.1±11.6ug/ml)). CRP was increased in 93% (6.2±4.4mg/dl), 84% had elevation of NTproBNP (551.7±434.0pg/ml). The ejection fraction (58.4±8%, 8%) was slightly decreased in 7 patients and WMS was increased in only 2 (1.16±0.22). Oedema (OS=0.29±0.08) and necrosis (NS=0.26±0.11) in all patients. The index of necrosis was 5.6%±1.7%. We found a significant correlation between: NTproBNP and WMS (R = 0.791 p = 0.002), NTproBNP and IN (R=0.756 p=0.02), and troponin and IN (R=0.688 p=0.005), CK and NS (R=0.580 p=0.017).

## Conclusions

In a patients with myocarditis, in spite of a preserved ejection fraction in the majority, the extent of oedema and necrosis evaluated by CMR correlated with the levels of serum biomarkers. CMR is a potential tool to define prognosis in these patients.

